# Machine learning reveals differential effects of depression and anxiety on reward and punishment processing

**DOI:** 10.1038/s41598-024-58031-9

**Published:** 2024-04-10

**Authors:** Anna Grabowska, Jakub Zabielski, Magdalena Senderecka

**Affiliations:** 1 Doctoral School in the Social Sciences, Jagiellonian University, Main Square 34, 30-010 Kraków, Poland; 2 Institute of Philosophy, Jagiellonian University, Grodzka 52, 31-044 Kraków, Poland

**Keywords:** Depression, Anxiety, Feedback processing, EEG, Machine learning, Cognitive control, Reward, Machine learning

## Abstract

Recent studies suggest that depression and anxiety are associated with unique aspects of EEG responses to reward and punishment, respectively; also, abnormal responses to punishment in depressed individuals are related to anxiety, the symptoms of which are comorbid with depression. In a non-clinical sample, we aimed to investigate the relationships between reward processing and anxiety, between punishment processing and anxiety, between reward processing and depression, and between punishment processing and depression. Towards this aim, we separated feedback-related brain activity into delta and theta bands to isolate activity that indexes functionally distinct processes. Based on the delta/theta frequency and feedback valence, we then used machine learning (ML) to classify individuals with high severity of depressive symptoms and individuals with high severity of anxiety symptoms versus controls. The significant difference between the depression and control groups was driven mainly by delta activity; there were no differences between reward- and punishment-theta activities. The high severity of anxiety symptoms was marginally more strongly associated with the punishment- than the reward-theta feedback processing. The findings provide new insights into the differences in the impacts of anxiety and depression on reward and punishment processing; our study shows the utility of ML in testing brain-behavior hypotheses and emphasizes the joint effect of theta-RewP/FRN and delta frequency on feedback-related brain activity.

## Introduction

Rewards and punishments powerfully shape human behavior. Impaired reward-seeking and avoidance of punishment have been systematically linked to various psychiatric disorders, especially depression. Multiple studies have reported that depressed individuals respond less strongly to rewarding stimuli than healthy individuals and are less likely to avoid punishments^1–3^.

The evidence for abnormalities in neural responses to rewards and punishments comes from reward positivity (RewP) and feedback-related negativity (FRN) in event-related potential (ERP) studies. RewP is a positive fronto-central wave that occurs approximately 250–350 ms after reward/positive feedback and is considered a neurophysiological marker of reward sensitivity^4^. Source localization and functional magnetic resonance studies link RewP amplitude with dopamine circuits^5,6^. Dopamine neurons play a key role in reward processing: when a reward is greater or worse than expected, the activation of these neurons increases or decreases, respectively^7,8^. Numerous studies have shown that RewP amplitude is attenuated in adults with depressive symptoms compared to non-depressed controls^9–12^. Proudfit and colleagues^13^ linked this amplitude attenuation with emotional disengagement, and thus blunted motivation and reward sensitivity in depressed individuals. A similar image emerges from a recent review conducted by Weinberg^14^. Based on a wide cross-section of works, Weinberg suggests that RewP might be a neural marker of deficits in attention and reactivity towards motivational content. Indeed, diminished RewP amplitudes were observed not only in clinically depressed individuals, but also in individuals with high self-reported anhedonia levels^1,10^. In turn, FRN is an ERP component that reaches its peak approximately 300 ms after punishment/negative feedback; it is usually observed as a larger negative deflection to loss trials relative to win trials. Larger magnitudes of FRN have been observed in both current and remitted depression^15,16^. Reward-related brain activity is often conceptualized as the difference between loss and win trials; in such cases terms *FRN* and *RewP* stand for difference waves and are often used interchangeably^4,17^. For a broader discussion of feedback-related ERPs, see^18^. Studies using this negative versus positive feedback conceptualization have reported blunted FRN/RewP amplitudes (i.e., a smaller negative minus positive feedback difference wave) in depressed versus control people^9,19^. However, abnormalities in reward and punishment sensitivity are not unique to clinical samples and have also been reported in general non-clinical populations with elevated depression-related symptoms^20–22^. Further, Umemoto et al.^23^ reported that healthy individuals who scored high in reward sensitivity and low in depression produced a larger RewP/FRN. These results suggest that changes in RewP and FRN amplitudes are likely to be expressed as a function of depressive symptoms, not simply as a binary change within formal diagnostic categories.

Although changes in the processing of both reward and punishment are observable in individuals with clinical depression and non-clinical elevated depression symptoms, in a recent study Cavanagh and colleagues proposed a more sophisticated dimensional hypotheses: depression is more associated than anxiety with reward/positive feedback processing (indexed with RewP), and anxiety is more associated than depression with punishment/negative feedback processing (indexed with FRN); thus, depressed individuals’ abnormal response to punishment may be related not to depression-specific symptoms but to anxiety symptoms^24^. Although symptoms of depression and anxiety often co-occur, there are actually some reasons to believe that they may be related to different circuits at the level of neuronal systems^25^. Reduced RewP has been found to be uniquely associated with depressive symptoms; no such relationship was found with anxiety symptoms^26,27^. Meanwhile, anxiety has been found to be associated with ERPs that share a common signature in the theta band: N2^28^ and error-related negativity (ERN)^29–31^. Although the FRN is also a theta-related ERP, no study reported an association between ERP FRN (understood as a response to negative feedback) and anxiety, as typically feedback-related ERPs have been quantified as negative versus positive feedback difference waves.

The aforementioned evidence for the associations between depression, anxiety, and feedback processing comes from ERP studies. However, time-frequency (TF) approaches to ERP activity have provided evidence that many ERP components contain a mix of overlapping TF components that index separable underlying processes. Theta (4-8 Hz) and delta (0-3 Hz) TF activity has been shown to underlie common ERP components, such as P3^32,33^, ERN^34,35^ and FRN^36–39^. Although closely related, feedback-P3 and FRN index different cognitive processes: FRN, which is usually linked with theta activity, represents reward sensitivity, while feedback-P3, which is linked with delta activity, represents the salience of reward^40^. For time-domain FRN and feedback-P3, Bernat and colleagues^38^ have shown that theta contributes to both increased negative amplitude of FRN and increased positive amplitude of P3. A slower delta contributes to a more positive amplitude of both the FRN and feedback-P3 components. Thus, the absolute amplitude of FRN (and RewP) is a combination of the ascending delta wave (most probably linked with the rising feedback-P3 component) and the descending theta wave. This complicates the estimation of the relative change in RewP/FRN magnitude since more negative RewP or FRN amplitudes could be the result of increased theta activity, blunted delta activity, or both. Further, although some studies link RewP to delta activity^24,36^, we believe this complicates the distinction between RewP and P3 components. Thus, we refer to RewP and FRN as to theta-related activities after positive and negative feedback, respectively, to highlight the separateness of these constructs from feedback-related brain activity in delta band^18^.

Indeed, not only ERPs but also time-frequency EEG activity are associated with reward processing. Multiple studies reported an increase in the high-beta/low-gamma oscillatory activity after rewarding feedback in gambling and learning tasks^41^. Specifically, the high-beta/low-gamma power activity in reward tasks was more pronounced for larger than smaller rewards but was not observed for worse-than-expected outcomes, which suggests the specific link between high-beta/low-gamma oscillatory and reward learning and attention^42–44^. Increased theta power in the frontal-medial brain area (FM$\theta$) has been observed for conflict, error processing^35^, and unexpected or negative outcomes^45–47^. Feedback-related FM$\theta$ has been linked to reward magnitude, with increased FM$\theta$ power for high compared to low reward magnitude^48^. This effect, however, has not been found to occur for participants with induced positive mood^49,50^, thus suggesting that optimistic bias leads to overestimation of the likelihood of positive events. Further, Cavanagh and colleagues^30^ linked increased FM$\theta$ with enhanced levels of anxiety. These results indicate that the affective or motivational state of participants influences reward processing, as indexed with FM$\theta$.

In the present study, we used a machine learning (ML) framework to address questions that emerged from Cavanagh and colleagues’^24^ research. As previously mentioned, they observed that depression predicts a significant reduction in RewP amplitude under the reward condition, while anxiety predicts an increased theta activity in the punishment condition. Based on these findings, we formulated the following questions: would high vs low depression groups be better differentiated by reward than punishment brain activity and would high vs low anxiety groups be better differentiated by punishment-theta than reward-theta brain activity? If the associations between anxiety, depression and neurophysiological measures are strong enough to translate into classification results, it would provide support for the Cavanagh and colleagues’ dimensional hypothesis. Such results would also be practically and clinically relevant. ML has repeatedly proven its usefulness and shown very good predictive performance^51^ in differentiating groups or conditions based on single-trial ERPs in various Brain-Computer Interface (BCI) tasks. It has also been used in various depression classification tasks and models for the diagnosis of major depressive disorder^52–55^. The strength of the ML approach lies in the data-driven extraction of the most discriminative brain signal features. Firstly, ML enhances the sensitivity of analysis. If groups actually differ in some brain signal characteristics, the ML model is likely to detect this difference. This sensitivity to differences decreases when only predefined features (e.g., the mean RewP amplitude recorded at one electrode within a selected time window) are taken into account. Secondly, ML identifies significant differences in brain signal activity between groups in a data-driven way. This provides a way to validate hypothesis-driven results by verifying whether the groups indeed differ as suggested by previous studies, and to discover new brain characteristics that are specific to some conditions and groups (e.g.^56^). The latter advantage falls within one of ML’s roles in neuroscience that was defined by Glaser and colleagues^57^: identification of predictive variables, i.e., identifying how informative one set of variables (e.g., neurophysiological) is about another (e.g., behavioral). This approach is still very rarely employed. The presented study aims to use ML in the aforementioned way to identify frequency-related brain signal features that discriminate between depression and control groups, as well as between anxiety and control groups; and to determine whether the features that differentiate depression from control groups are the same or different from those that differentiate anxiety from control groups. Further, the ML framework offers increased generalizability of the results compared to classical statistical approaches in between-group studies^58^. For a discussion on the utility of ML models in neuroscience, see^57^.

To take full advantage of the benefits of ML, we used the Common Spatial Pattern (CSP) algorithm for brain signal feature extraction^59^. Classic ERP approaches are based on the signal from single electrodes or pre-defined regions of interest. Single-electrode EEG signals are characterized by a low signal-to-noise ratio, thus predictive analyses based on these signals entail a high risk of overfitting and low model sensitivity^59^. Feature extraction methods increase the signal-to-noise ratio and extract information on the brain spatial dynamics, which is rarely analyzed^60^. In recent decades several methods have been proposed for EEG signal features extraction. Some of the most popular methods are: the wavelet transform method^61^, independent component analysis^62^, and principal component analysis^63^. These methods, categorized as unsupervised, extract features based on data variance, i.e., the distribution of information within the data, which may lead to the loss of valuable information^64^. The CSP algorithm, as a supervised ML method, follows a very different strategy. The goal of CSP is to find a set of spatial filters that can effectively differentiate between two classes of signals based on their covariance matrices; thus, CSP is specifically designed for classification. Before the deep learning era, CSP was the most popular and most successful method in the classification of EEG signals^65^. CSP was originally used to detect abnormal EEG signals, but it has also been successfully used in BCI tasks^65–68^. An additional advantage of CSP is the reduction of the signal-to-noise ratio; which in turn diminishes the sensitivity of the model to non-task-related changes in the measured signals^59^. Thus, CSP-based classification models show considerably higher accuracy, generalizability and reliability than classification based on classic ERP features^69,70^.

Based on the aforementioned literature, we hypothesized that it would be possible to predict depression vs. control group membership based on feedback-related brain activity, as the literature suggests that depression alters feedback processing. To broaden the knowledge on the relationships between depression, anxiety, and feedback processing, we additionally aimed to test three more specific hypotheses: (1) due to impairment of the motivational approach system (linked with the dopamine system) and the consequently altered positive feedback processing, classification of individuals with high severity of depression symptoms vs controls will be more accurate when it is based on the reward-theta than when based on punishment-theta activity; (2) as theta activity has been found to be enhanced in loss trials in individuals with high severity of anxiety symptoms vs controls in both clinical and nonclinical populations^31^, classification of individuals with high severity of anxiety symptoms will be more accurate when it is based on the punishment-theta than when based on reward-theta activity; (3) as some of the variability in the classical RewP/FRN ERP components is induced by the positive delta-P3 wave, it will be possible to predict depression vs. control group membership based on reward-delta or/and punishment-delta activity. Particularly, we were interested in whether the delta changes in the RewP/FRN (200-300 ms) time window are already so significant as to distinguish experimental and control group membership. If so, this would be further evidence of an overlap between various TF components and the confounding effect of this overlap on study results.

To emphasize the utility of ML and supervised brain signal feature extraction, we conducted additional classic ERP analyses. We expected that depressed vs control individuals should be better differentiated when based on reward-related Rew-P rather than punishment-related FRN; anxious vs control individuals should be better differentiated based on punishment-related FRN rather than reward-related Rew-P. However, considering RewP/FRN (theta) and P300 (delta) brain signal entanglement, we assumed that these relationships may not be robust enough to yield statistically significant results.

## Methods

### Participants

Two hundred and twenty-five volunteers (113 females, 111 males, one non-binary gender) aged 18-39 (*M* = 23.64, *SD* = 4.18) took part in the study. Participants were recruited from the general population via internet advertisements. All participants were healthy, free of medications, declared no history of neurological or psychiatric diseases, and had normal or corrected-to-normal vision. The participants’ average number of years of education was 15.41 (*SD* = 2.40). To ensure the highest quality of EEG data, each segment containing artifacts on any of the set of 64 electrodes was discarded. As a result, three participants who performed less than five artifact-free trials that contained positive feedback and less than five artifact-free trials that contained negative feedback were excluded prior to analysis.

Participants were assigned to the groups based on their scores in the Beck Depression Inventory-II (BDI-II)^71,72^ and the State-Trait Anxiety Inventory, trait subscale (STAI-T)^73,74^. The BDI-II is administered to both clinical and non-clinical populations, with a clinical cut-off of 13. The STAI-T has no defined clinical cut-off, but a cut-off of 40 or 41 is most commonly used to determine the probable clinical level of anxiety^75^. Based on these cut-offs, participants were divided into four groups: high BDI-II and high STAI-T (DEP; BDI-II > 13; STAI > 41); low BDI-II and high STAI-T (CTR DEP; BDI-II ≤ 13; STAI > 41); low BDI-II and high STAI-T (ANX; BDI-II <= 13; STAI > 42); low BDI-II and low STAI-T (CTR ANX; BDI-II ≤ 13; STAI < 41). The mean BDI-II score in the depressive (high BDI-II and high STAI-T; *M* = 23.47) and control (low BDI-II and high STAI-T; *M* = 7.07) groups corresponded to the results of a psychometric study of the Polish adaptation of the BDI-II (control group: *M* = 9.89; mild depression group: *M* = 22.94; severe depression group: *M* = 31.29). Due to the significant overlap between anxiety and depressive symptoms, it was impossible to isolate a purely depressive group; therefore, to minimize the impact of anxiety symptoms, we chose to keep anxiety high in both the depressive and control groups. There were no behavioral differences between groups associated with the number of positive and negative feedback received or performance (p > 0.09 for all group comparisons). Table 1 summarizes the characteristics of the participants within groups.

**Table 1:** Demographic, symptom and behavioral characteristics by group.

		DEP	CTR DEP	ANX	CTR ANX
Depression (BDI-II)	*M* (*SD*)	23.47 (8.86)	07.07 (3.68)	07.02 (3.68)	04.44 (3.31)
	Min/Max	14/48	0/13	0/13	0/12
Anxiety (STAI-T)	*M* (*SD*)	54.11 (6.24)	47.19 (3.55)	47.75 (3.27)	34.89 (4.21)
	Min/Max	42/70	42/55	43/55	25/40
Age (years)	*M* (*SD*)	23.08 (3.45)	23.36 (4.28)	23.43 (4.26)	24.39 (4.64)
	Min/Max	18/38	18/39	18/39	19/39
Education (years)	*M* (*SD*)	15.47 (2.37)	15.06 (2.08)	15.05 (2.14)	15.88 (2.79)
	Min/Max	11/25	9/20	9/20	12/24
Number of positive feedback	*M* (*SD*)	86.45 (28.71)	94.35 (27.41)	94.06 (28.28)	88.95 (29.69)
	Min/Max	31/139	20/169	20/169	22/150
Number of negative feedback	*M* (*SD*)	135.13 (27.64)	128.35 (27.52)	128.70 (28.45)	134.17 (29.82)
	Min/Max	83/192	54/204	54/204	70/202
Number of error trials	*M* (*SD*)	32.88 (14.16)	31.42 (14.86)	30.17 (14.31)	29.33 (13.93)
	Min/Max	2/72	7/77	7/77	3/70
Number of inhibited trials	*M* (*SD*)	79.12 (14.16)	80.58 (14.86)	81.83 (14.31)	82.67 (13.93)
	Min/Max	40/110	35/105	35/105	42/109
Gender	F/M/NB	44/31/0	40/31/1	36/28/1	24/42/0
*N*		75	72	65	66

*M* mean, *SD* standard deviation, *F/M/NB* female/male/non binary gender, *DEP* depression group, *CTR DEP* control for depression group, *ANX* anxiety group, *CTR ANX* control for anxiety group.

### Procedure and task

**Figure 1:**
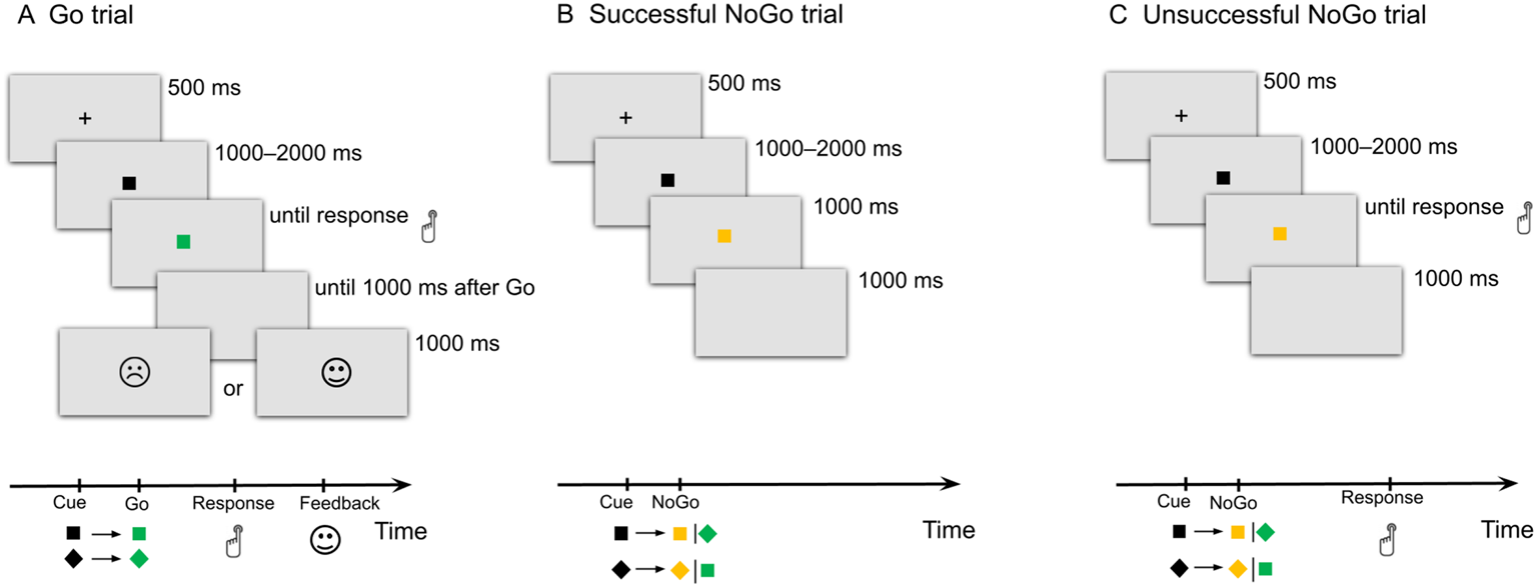
Scheme of the Go/NoGo task used and its conditions: Go trials (**A**); successful NoGo trials (**B**); unsuccessful NoGo trials, i.e., erroneous response (**C**).

While the EEG signal was recorded, participants performed a speeded version of the Go/NoGo task that has previously been validated in several studies^76–78^. The task included a training block (15 trials), four experimental sessions of 84 trials each, and two calibration blocks of 14 trials, which preceded the first and third experimental blocks. Participants had to press the response key as quickly as possible if the black geometric figure (square or diamond) turned green and kept the same spatial orientation (two-thirds of the trials, all corresponding to Go trials). By contrast, they were asked to withhold their response if the black figure turned green but changed orientation, or if it turned orange irrespective of orientation (one-third of the trials, all corresponding to NoGo trials). On each go trial, the RT was compared against an arbitrary cut-off. If the RT speed was above the cut-off limits (slow-Go trials), then negative feedback (a sad face) was provided 1,000 ms after target onset. In turn, if the RT speed was below the cut-off limits (fast-Go trials), positive feedback (a smiling face) was presented. To receive positive feedback during the first two experimental blocks, participants had to be 10% faster than the mean RT calculated during the first calibration block; during the third and fourth experimental blocks, participants had to be respectively 10% and 20% faster than the mean RT calculated during the second calibration block. The RT cut-off was determined for each participant separately, without his/her knowledge, and was adjusted during the experimental session to overcome the interindividual variability in the speed of motor responses and to deal with the effects of time and learning. No feedback was provided after inhibitory errors and successful response inhibition in NoGo trials to enhance internal monitoring in these cases. Figure 1 presents an outline of the task.

The study followed the Declaration of Helsinki^79^, and the protocol was approved by the Research Ethics Committee of the Philosophical Faculty of Jagiellonian University in Kraków, Poland. All participants received verbal and written information about the study’s purpose and procedure; they also provided written informed consent and were monetarily compensated for their time.

### Electrophysiological recording and data pre-processing

**Figure 2:**
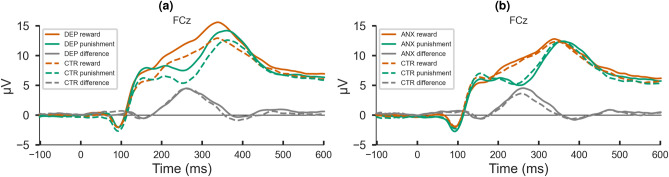
Positive-feedback, negative-feedback, and positive minus negative feedback ERP waveforms for depression and control groups (**a**) and anxiety and control groups (panel B). The visible difference between depression and control groups in condition waves is no longer present in the conditions difference wave. This suggests that the difference between depression and control groups in RewP/FRN amplitudes in condition waves is not driven by RewP/FRN (panel A). The visible difference between anxiety and control groups in the conditions difference wave suggests that the difference between anxiety and control groups in RewP/FRN amplitudes in condition waves is driven by RewP/FRN (**b**).

The experiment was conducted by trained researchers in a sound-attenuated room. The EEG signal was continuously recorded at 256 Hz from 64 silver/silver-chloride (Ag/AgCl) active electrodes (with preamplifiers) using the BioSemi Active-Two system and referenced online to the CMS-DRL ground, which drives the average potential across all electrodes as closely as possible to amplifier zero. The horizontal and vertical electro-oculograms (EOGs) were monitored using four additional electrodes placed above and below the left eye and in the external canthi of both eyes after adequate skin preparation. The EEG signal was pre-processed with BrainVision Analyser Software^80^. The signal was re-referenced off-line to the average of the left and right mastoid electrodes; it was initially filtered with a Butterworth fourth-order filter with a high pass of 0.05 Hz, and a Butterworth second-order filter with a low pass of 30 Hz. Power-line noise was removed with a notch filter at 50 Hz. Data were further epoched around the feedback onset (-1,000 to 2,400 ms) and baseline corrected to the average activity from -250 to 0 ms pre-feedback. The ocular artifact correction was performed with Gratton, Coles, and Donchin’s algorithm^81^. Noise epochs were rejected via an automatic procedure with the AutoReject MNE package^82^. The average number of artifact-free positive-feedback epochs included in the analysis per participant was 83.30 (*SD* = 27.56); for negative-feedback epochs, it was 121.34 (*SD* = 30.41). Further, epochs were averaged by condition to yield positive-feedback and negative-feedback ERPs for each participant. Figure 2 shows condition ERPs along with the positive minus negative feedback difference waves per group.

### Data reduction and analysis

#### Time-domain components: RewP and FRN

 The classic reward-locked Rew-P and punishment-locked FRN ERP components were quantified as the mean activity from 200 to 300 ms at electrode FCz in the positive-feedback and negative-feedback ERPs, respectively. It should be noted that, unlike previous studies that analyzed RewP/FRN as the difference wave between positive and negative feedback ERPs^9^, in the current study RewP and FRN were calculated separately.

#### Time-frequency components: theta and delta

**Figure 3:**
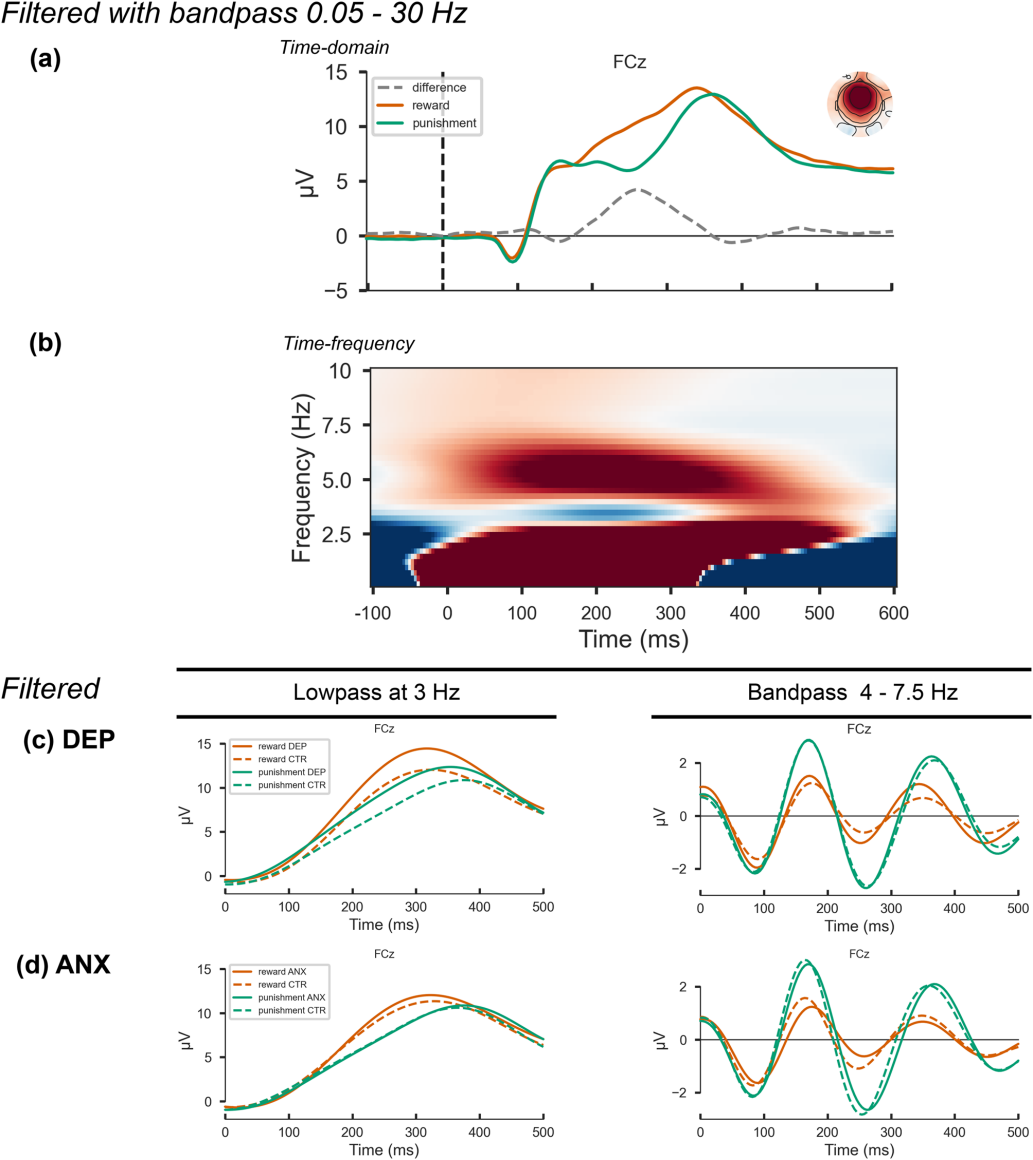
Time-domain and time-frequency (TF) decomposition of feedback-locked ERPs. All ERPs and TF representations are plotted on the FCz channel. Row (**a**), waveform plot: averaged unfiltered reward and punishment feedback-locked ERPs across all trials, filtered with 0.05–30 Hz band-pass. The headmap shows the RewP-FRN difference topography at 250 ms. Row (**b**), TF-representation plot: averaged TF representation of the ERP across all trials and conditions. Row (**c**) and (**d**), waveform plots: averaged reward and punishment feedback-locked ERP activity across all trials, frequency-filtered using FIR filters to isolate lower-frequency (3 Hz lowpass) and higher-frequency (4–7.5 Hz bandpass) activity for the depression and control groups (**c**); anxiety and control groups (**d**).

 Following the methodology outlined in^36^ that has been shown to be effective in separating delta and theta bands in feedback-related ERPs, the feedback-locked ERPs were filtered using a separate high-pass FIR filter at 3 Hz (non-causal FIR low-pass filter with windowed time-domain design, upper transition bandwidth: 2.00 Hz (-6 dB cutoff frequency: 4.00 Hz), hamming window with 0.0194 passband ripple and 53 dB stopband attenuation) to isolate delta frequency, and a bandpass FIR filter at 4 to 7.5 Hz (non-causal FIR bandpass filter with windowed time-domain design, lower transition bandwidth: 2.00 Hz (− 6 dB cutoff frequency: 3.00 Hz), upper transition bandwidth: 2.00 Hz (− 6 dB cutoff frequency: 8.50 Hz), hamming window with 0.0194 passband ripple and 53 dB stopband attenuation) to isolate the theta frequency. The theta and delta filter cutoffs were chosen based on visual inspection of the averaged TF energy representation across all subjects and feedback (Fig. 3b), filtered with 0.05–30 Hz Butterworth band-pass filter. Theta and delta components were further narrowed to a time window of 200-300 ms and filtered with CSP. The aim of CSP is to maximize the variance of the spatially filtered signal under one condition while minimizing it for the other condition; thus, it is widely used in classification tasks for data dimensionality reduction in the spatial domain. The result of CSP is a set of spatial patterns that most differentiate the defined groups. Extracting these patterns allowed us to determine what type of activity (delta, theta) in which brain areas most differentiated the experimental group from the control groups.

#### Evaluation

 We conducted separate classifications for each unique combination of (1) EEG components: delta, theta, ERP; (2) feedback valence: positive, negative; (3) the anxiety and depression groups. The classification task was performed using the Support Vector Classifier (SVC). The hyperparameters of the models included the number of CSP components (1–4) and the CSP regularization parameter $\lambda$ ($10e^{-3}$–$10e^{-1}$) when applicable; the SVC kernel (linear, radial); and the SVC regularization parameter *C* ($10e^{-5}$–$10e^1$). The models were trained using threefold cross-validation (CV). Cross-validation is a resampling procedure used to estimate the efficiency of an ML model on unseen data. It facilitates the estimation of the expected general performance of the model when used to make predictions on data not used during the training of the model. Application of cross-validation results in a less biased and/or less optimistic estimate of the model efficiency in comparison to a simple train/test split. Due to the small amount of data, no external test set was used; the best hyperparameters of SVC were chosen based on the mean of cross-validated scores; fitted models were compared in terms of 10-repeated tenfold (10 × 10) CV scores^83^. The statistical significance of the results was assessed with permutation tests^84^. Model comparison was performed with Nadeau and Bengio’s corrected paired t-test, which is recommended for comparison of ML models^83^.

## Results

### EEG features

There were significant effects of feedback type on ERP, delta and of theta mean amplitude at FCz in the selected time window (200–300 ms) for all groups (all p < 0.001). The grand averages of the time-domain RewP and FRN waveforms are shown in Fig. 3a. Grand averages of the theta and delta waveforms for all four groups are shown in Fig. 3c,d.

The use of CSP provides insight into the spatial areas that best differentiate experimental and control groups. In the classification of depression, our theta-based models highlighted frontal brain areas. This is consistent with the expectation that predominantly frontal theta would be associated with the severity of depressive symptoms. In the delta-based models, CSP distinguished and highlighted the central and parietal areas of the brain. For the anxiety classification task, the theta-based models did not reveal specific strong spatial clusters that are consistent for both reward and punishment brain activity: a weak focus on the frontal area is visible for the reward-theta model, while the punishment-theta model primarily accounted for the signal at the Cz electrode. In the delta-based models, CSP highlighted a strong centro-parietal cluster in the punishment-related brain activity; in the reward-related brain activity, there is no visible focus on any specific brain area. The CSP spatial patterns for the delta- and theta-based depression and anxiety classifications are shown in Supplementary Fig. S1. Visualizations of the differences between the experimental DEP/ANX and control groups in delta/theta activity for reward and punishment feedback along with group differences in CSP spatial patterns are shown in Supplementary Figs. S2 and S3 for depression and anxiety classification, respectively.

### Classification results: Time-frequency components

**Figure 4:**
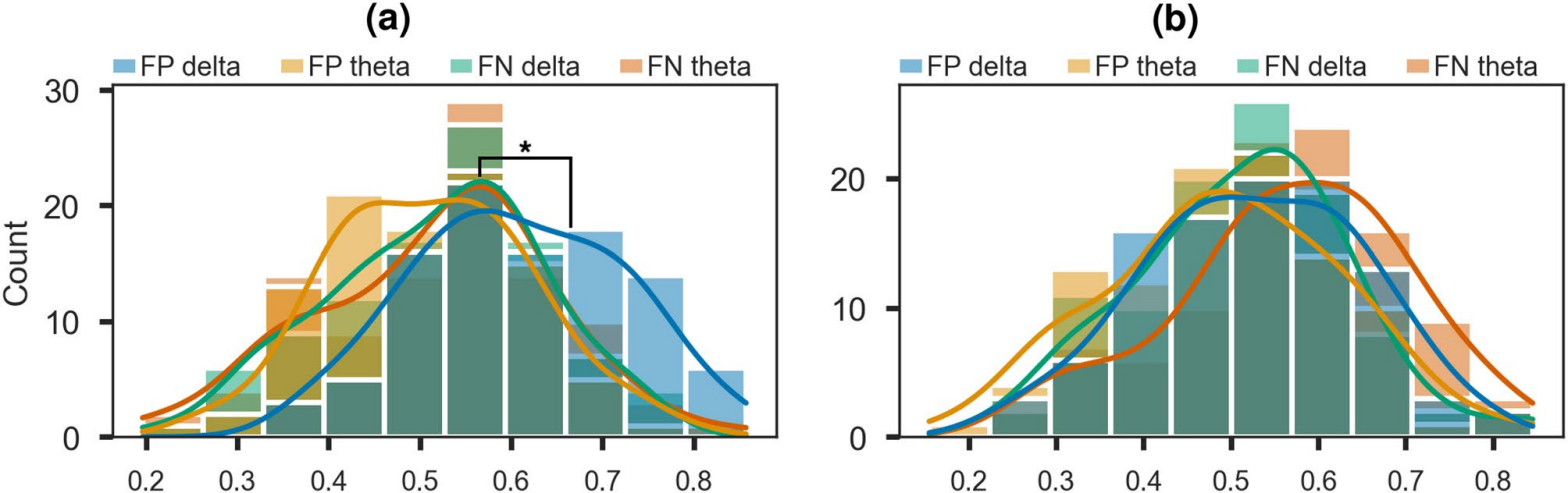
Post hoc analysis of differences between the reward and punishment models. Distribution of 10 × 10 CV balanced accuracy scores for depression vs control classification models (**a**) and anxiety vs control classification models (**b**). FP = positive/reward feedback; FN = negative/punishment feedback. Significant differences between distributions are marked. Significance levels: *$p<0.05$.

It was possible to predict membership of the depressed versus control group based on feedback-related brain activity. The reward-delta, punishment-delta, and reward-theta classifiers yielded significant results (p$_{ACC} <0.050$), with the mean cross-validated balanced accuracy ranging from .56 to .64. The punishment-theta classifier did not achieve the significance threshold (p$_{ACC}$ = .100). *Post hoc* direct comparison of the reward- and punishment-based models with Nadeau and Bengio’s corrected paired t-test revealed that the mean performance of the reward-delta model (ACC = 0.61; ROC AUC = 0.65) was significantly better than the mean performance of the punishment-delta model (ACC = 0.52; ROC AUC = 0.52; p$_{ACC}$ = 0.042; p$_{RAUC}$ = 0.021). There was no difference in accuracy scores between the reward- and punishment-theta-based models (p$_{ACC}$ = .464; p$_{RAUC}$ = .302). Density plots of the balanced accuracy scores, tested for differences with Nadeau and Bengio’s corrected paired t-test, are shown in Fig. 4a. For anxiety versus control classification, all but the punishment-theta classifiers yielded significant results (p$_{ACC}$ < 0.050; p$_{ACC}$ = 0.108 for the punishment-theta model), with the mean cross-validated balanced accuracy ranging from 0.57 to 0.61. *Post hoc* comparison of the reward- and punishment-based models revealed that they did not differ from each other in terms of balanced accuracy scores (p$_{ACC}$ = 0.405; p$_{ACC}$ = 0.141 for delta and theta respectively). However, the mean ROC AUC of the punishment-theta model (ROC AUC = 0.61) was significantly greater than the mean ROC AUC of the reward-theta model (ROC AUC = 0.47; p$_{RAUC}$ = 0.054). This difference, although it is insignificant, is also visible in the histograms of balanced accuracy scores for the anxiety models (Fig. 4b; the difference between FN and FP theta). A summary of the depression and anxiety models’ performance is shown in Table 2. The results of the *post hoc* comparison of reward- and punishment-based models for ROC AUC, precision, and recall metrics can be found in Supplementary Fig. S4.

**Table 2:** Detailed results of depression and anxiety models based on delta, theta, and ERPs.

	Depression						Anxiety					
	Train ACC	ACC	*p*-value	R-AUC	Recall	Precision	Train ACC	ACC	*p*-value	R-AUC	Recall	Precision
FP delta	0.67	**0.64**	0.003	0.65	0.63	0.66	0.69	**0.58**	0.032	0.54	0.72	0.56
FN delta	0.80	**0.60**	0.011	0.56	0.64	0.61	0.57	**0.60**	0.015	0.63	0.68	0.60
FP theta	0.62	**0.60**	0.006	0.58	0.75	0.59	0.68	**0.61**	0.006	0.57	0.67	0.59
FN theta	0.72	0.56	0.101	0.51	0.53	0.57	0.63	0.57	0.109	0.58	0.58	0.57
RewP	0.55	0.53	0.319	0.51	0.53	0.55	0.50	0.48	0.795	0.42	0.03	0.11
FRN	0.56	0.54	0.221	0.57	0.53	0.55	0.50	0.51	0.340	0.49	0.14	0.17

Findings with *p*-value below 0.05 are shown in bold.

FP = positive/reward feedback; FN = negative/punishment feedback; RewP = reward positivity; FRN = feedback-related negativity; ACC = balanced accuracy; R-ACC = ROC AUC. With the exception of Train ACC, all scores reported are the average of threefold cross-validation. Train ACC stands for balanced accuracy score estimated on the entire dataset, without cross-validation.

We visualized CSP delta/theta activity differences between groups after reward and punishment feedback. Participants with high severity of depressive symptoms exhibited increased reward-theta activity compared to the control group. They also displayed increased reward and punishment delta activity compared to the control group. The anxiety group showed diminished reward-related theta activity compared to the control group; it also displayed diminished reward- and punishment-related delta activity compared to controls.

### Classification results: time-domain ERP components

It was not possible to predict membership of the depressed or the anxiety group based on the RewP or FRN. Similar to the main analyses, classification was conducted using the SVC classifier and threefold cross-validation to improve the generalizability of results. None of the created depression and anxiety models yielded significant results. The mean balanced accuracies of the RewP-depression model and the FRN-depression model were 0.53 (p$_{ACC}$ = 0.319) and 0.54 (p$_{ACC}$ = 0.221), respectively. Direct comparison of the RewP-based and FRN-based models for depression classification revealed that neither RewP nor FRN showed a significant advantage in the classification of depression (p$_{ACC}$ = 0.238; p$_{RAUC}$ = 0.319). For the anxiety classification, the mean balanced accuracy of the RewP model was 0.48 (p$_{ACC}$ = 0.795), while for the FRN model it was .51 (p$_{ACC}$ = 0.340). There was no significant difference in performance between the FRN-based and RewP-based models (p$_{ACC}$ = 0.459; p$_{RAUC}$ = 0.383). We performed additional exploratory analyses with P3 amplitude control, due to the problem of mixing RewP/FRN and P3 signals. None of the models created were significant, but the quality of the models for depression slightly improved. The results of the exploratory analysis can be found in Supplementary Table S1.

In summary, the results of the analysis indicate the limited ability of the models to accurately identify and classify depression or anxiety versus controls. Further research and model improvements are needed to increase classification performance in the ERP area.

## Discussion

The aim of the present study was to use ML predictive modeling to test the possibility of predicting depression vs. control group membership based on feedback-related brain activity. Additionally, we aimed to test the dimensional hypothesis that depression is associated with impaired processing of positive feedback; anxiety, which is comorbid with depression, is associated with impaired processing of negative feedback. Toward this aim, we separated feedback-related brain activity into delta activity driven by the P3 wave (later called P3-delta) and theta activity related to FRN/RewP. Additionally, we were interested in whether the difference between the depression/anxiety and control groups was driven more by P3-delta, or by FRN/RewP-theta punishment/reward activity.

It was possible to predict membership to the group with high severity of depressive symptoms versus the control group based on feedback-related brain activity; this is consistent with previous non-ML works which reported that altered feedback processing is associated with high severity of depressive symptoms^10,19,22,24^. Our study is the first to show not only that there is a statistically significant difference in feedback-related brain activity between the depression and control groups, but also that it is possible to successfully predict group membership based on feedback-related brain activity. Importantly, successful prediction was only possible with the CSP spatial filter, as depression vs control classification was not possible based on the classic ERP RewP and FRN components. In the classic ERP analysis, dimensionality reduction is usually based on the selection of a single electrode that greatly reduces the variability of the brain signal; this may limit the level of generalizability of brain-behavior models. Thus, CSP-based classification is a powerful tool for testing whether there are indeed differences between two or more groups.

We observed that participants with high severity of depressive symptoms exhibited enhanced reward-theta activity that attenuated ERP RewP amplitude; this finding is consistent with the existing literature^15,16^. Participants also showed enhanced reward- and punishment-delta activity that made the ERP RewP and FRN amplitudes more positive. Since the influence of delta activity was much stronger than theta activity, without delta and theta separation one could conclude that depression is linked with enhanced ERP RewP amplitude. Our results clearly showed that although the overall RewP amplitude in depressed participants was greater than in controls, this difference was driven by the P3-delta wave; the theta-related differences between the groups indicated reduced ERP RewP amplitude in depressed participants. Participants with high levels of anxiety symptoms were characterized by reduced reward-theta activity that enhanced ERP RewP amplitude. Similar results have been reported in studies on social anxiety^85^; this suggests that specific symptoms of anxiety may affect the processing of positive feedback and alter the motivational system. Compared to controls, participants with high levels of anxiety exhibited slightly reduced punishment-theta activity that attenuated the negative amplitude of FRN and thus enhanced overall ERP FRN amplitude. However, this effect was marginally significant. Anxiety was also associated with modestly blunted reward- and punishment-delta activity that made the overall ERP RewP and FRN amplitudes more negative. The FRN ERP amplitudes were products of two activities acting in opposite directions: theta activity, which made ERP FRN amplitude more negative; and delta activity, which made ERP FRN amplitude more positive. Thus, differences between reward-related RewP and punishment-related FRN amplitudes in anxiety may be driven by changes not only in RewP/FRN-theta activity but also in P3-delta activity.

Based on the results of the classification and post hoc tests, we cannot confirm the dimensional hypothesis on the differential effects of depression and anxiety on reward and punishment processing as indexed with RewP and FRN^24^. In our study, the visible and significant difference between the depression and control groups was driven mainly by P3-delta activity; there were no differences between reward- and punishment-theta activities. The high severity of anxiety symptoms was marginally more strongly associated with punishment feedback processing than with reward feedback processing; this may suggest a dissociative effect of anxiety on the processing of rewards and punishments that is consistent with our expectations but is only mildly detectable in our groups. The absence of a conclusive result does not necessarily indicate that the dimensional hypothesis is false: it should be noted that our groups differed not only in terms of relevant symptoms (BDI-II for depression; STAI-T for anxiety) but also in terms of comorbid symptoms, and the analyses were performed on a non-clinical sample. However, the differences in comorbid symptoms were much smaller than the differences in relevant symptoms, therefore we expected them to also be smaller at the models’ performance level. It should be noted that when testing the dimensional hypothesis we limited our frequency of interest to theta activity as we consider reward-theta and punishment-theta to be representations of the RewP and FRN components, respectively. In the study by Cavanagh and colleagues^24^, reward-related RewP was defined as reward-delta activity. Under this framework, we would confirm Cavanagh’s hypothesis of an association between reward-delta and depression, as we found a significant difference between the depression and control groups in reward-delta but not in punishment-delta activity. Nonetheless, we are inclined to attribute delta activity to the P3 wave, which is associated with sensitivity to the amount of reward, not with the degree to which an individual’s behavior is motivated by rewarding/punishing stimuli, which is indexed by theta-related RewP/FRN^18^.

Our results showed a joint effect of the RewP/FRN and P3 components on brain activity after receiving feedback in the early 200-300 ms time window. This highlights the importance of theta and delta separation to reliably test hypotheses related to feedback processing. In our study, the visible difference between the group with high levels of depressive symptoms and the control group was primarily driven by P3-delta activity. This emphasizes the need to precisely define the RewP and FRN components and take into account the effect of P3 on the waveform of feedback-related brain activity in order to improve the reliability and comparability of results.

At least four issues limit the results and conclusions of the presented study. The most important caveat of the presented study is the limited variability of the analyzed anxiety and depressive symptoms as participants with any mental disorder were excluded. Our dataset certainly did not cover the entire spectrum of severity of anxiety and depressive symptoms, therefore the study’s conclusions are inherently limited. It is possible that the associations between feedback processing, anxiety, and depression change their pattern in the clinical population, or maybe our analysis did not capture some of the important clinical relationships due to the healthy volunteer population’s generally low levels of symptoms. In addition, our experimental and control groups differed in more than one symptom. Since the differences in relevant symptoms were much larger than in non-relevant ones, we assumed that it was the difference in relevant symptoms that led to the difference between the models’ performance. However, we cannot exclude the possibility that the results of our analysis were distorted by differences in the severity of comorbid symptoms. The dimensional hypothesis needs to be further tested on pure groups. Further, categorical depressed/anxious versus control contrasts are dependent on an arbitrary cut-off point. Splitting groups based on BDI-II and STAI measures is associated with a reduced power to detect effects as it removes information about the full range of severity within the sample. It also gives a special significance to the cut-off value which may or may not reflect a valid, non-linear category, especially in our population of healthy individuals. Although we ensured that our depression and control groups were representative of symptom severity levels as established within the Polish adaptation of the BDI-II questionnaire, it should be noted that participants did not receive a diagnosis of depression or anxiety. Thus, the results of our categorical approach should be interpreted with caution in the clinical context. Finally, due to an insufficient amount of data, we did not separate the external test set, therefore our models were compared only in terms of 10 × 10 CV scores. This limits the level of generalizability of our models. It would be beneficial to conduct similar analyses on a larger dataset and test the level of generalizability of models on a separate testing set. Future studies would benefit from adopting a continuous approach that models the full range of severity and controls for confounding variables. In addition, since several oscillatory components in the theta, alpha, and high-beta/low-gamma bands have been shown to be associated with rewards and losses, future studies may assess the effects of depression and anxiety not only on delta and theta but also on higher brain frequencies.

In summary, our study shows the utility of ML in testing brain-behavior hypotheses. Using the CSP spatial filter, we revealed patterns of brain activity that differed between the depression/anxiety and control groups. We also demonstrated that on the basis of these patterns it was possible to create models that achieved higher accuracy than models based on classic ERP features. Finally, we showed the joint effect of theta-RewP/FRN and delta-P3 on feedback-related brain activity, which can distort results.

### Supplementary Information

Supplementary Information.

## Data Availability

The additional online resources and the dataset containing the EEG recordings and questionnaire data are available in the *BluishO* repository, https://osf.io/f2a86/. The detailed results and the code for reproducing the analyses from the presented study are available at https://github.com/abelowska/bluishO under the MIT License.
