# Supplementary material for: Machine learning reveals differential effects of depression and anxiety on reward and punishment processing
Source: Sci Rep. 2024 Apr 10;14:8422. doi: 10.1038/s41598-024-58031-9 (PMC11366008; doi:10.1038/s41598-024-58031-9)
Supplement: Supplementary file 1 — Supplementary Information. [file 41598_2024_58031_MOESM1_ESM.pdf]

Spatial patterns of the CSP components

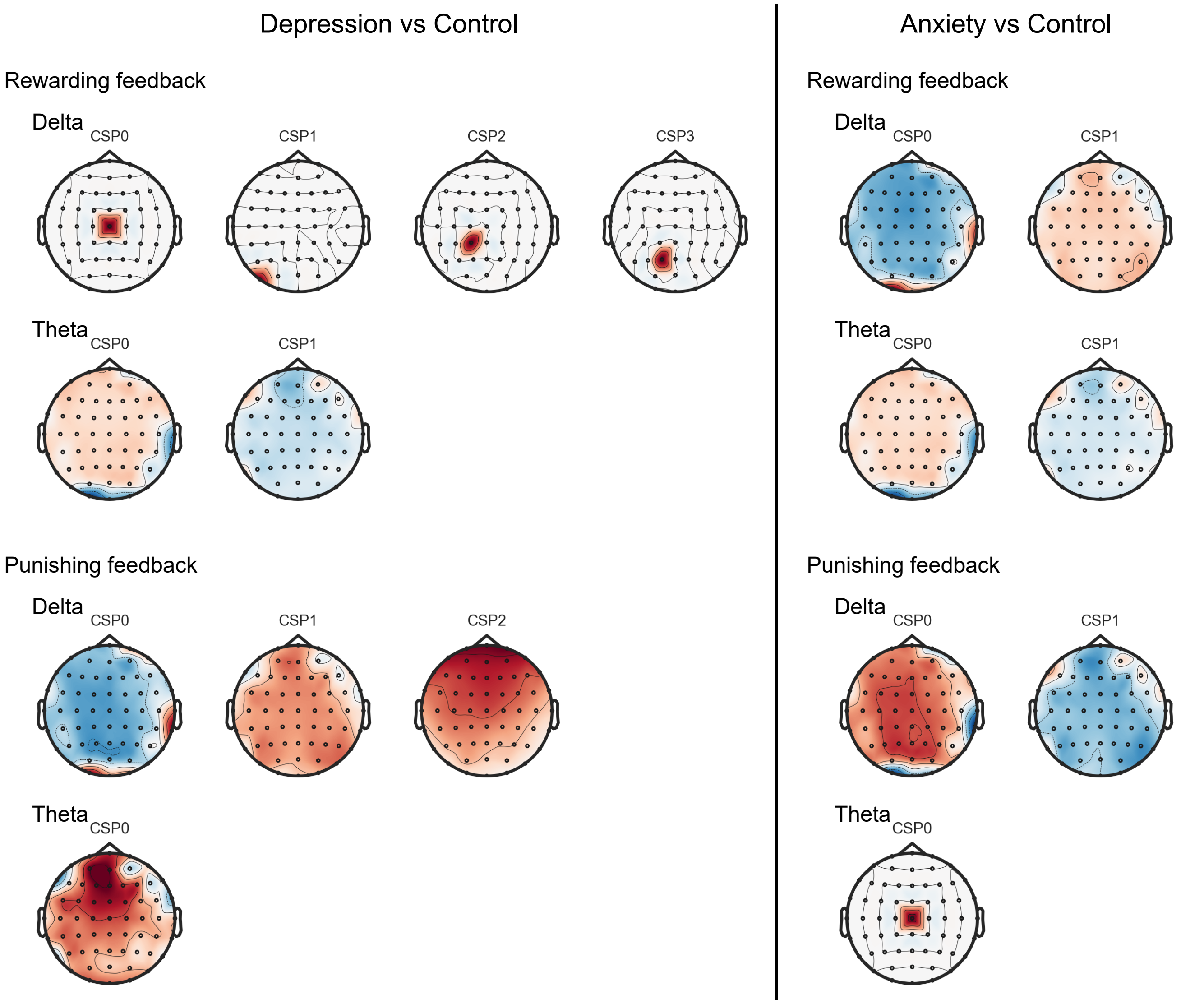

Supplementary Figure S 1. Spatial patterns of the CSP components. The number of CSP components was selected automatically during hyperparameter tuning. The CSP components maximize the variance in brain signals between groups, thus the extracted components highlight the specific brain activity that differentiates the groups. There is a visible focus on the frontal areas of the brain in the theta-based models, except for the punishment model for anxiety classification, where mainly the signal on the Cz electrode was taken into account.

Experimental vs control groups spatial pattern differences

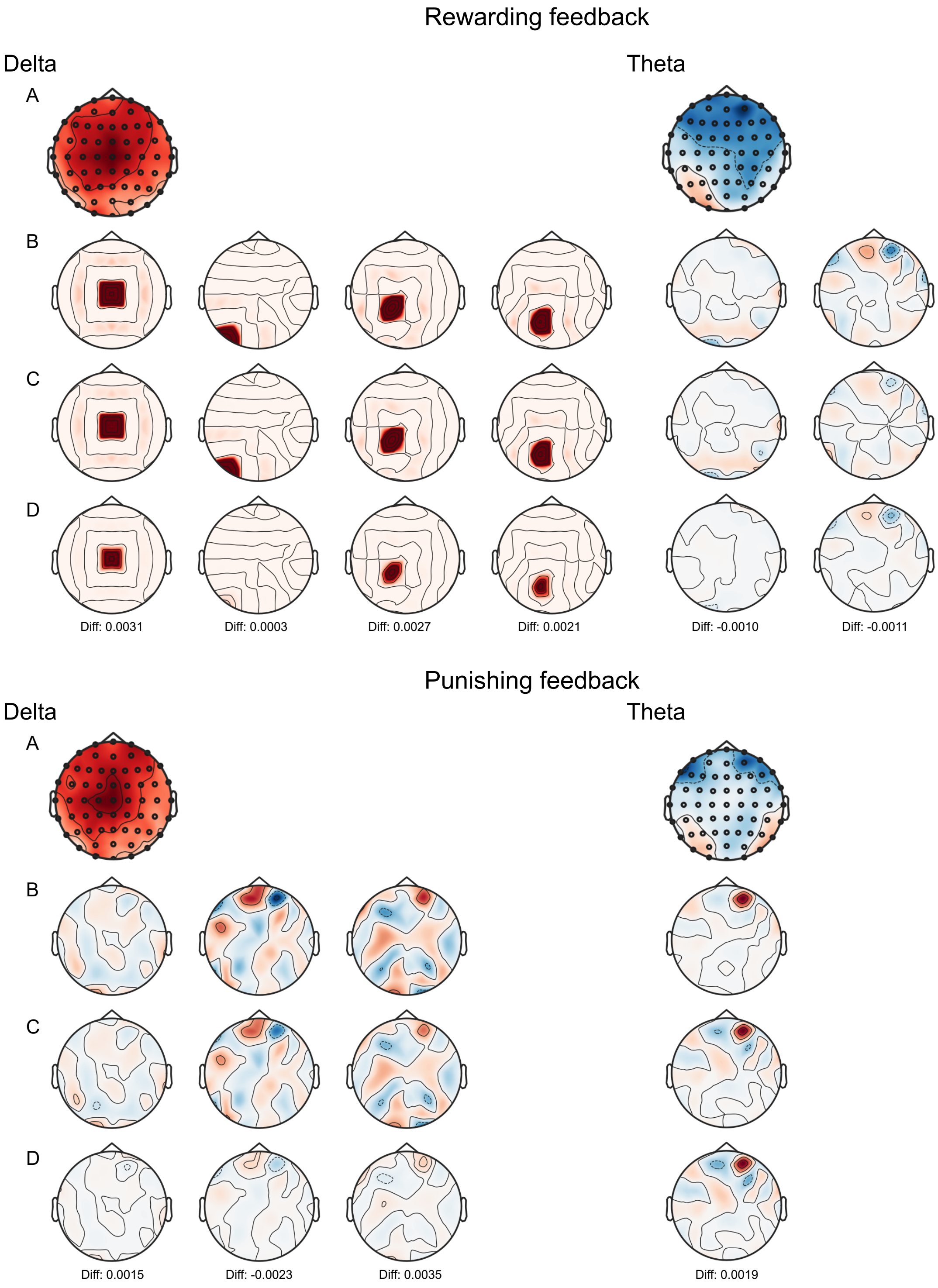

Supplementary Figure S 2. Depression vs control group differences. Difference between depression and control groups in the frequency domain, before CSP transformation (A). CSP spatial patterns for the depression group (B). CSP spatial pattern for the control group (C). Difference between depression and control groups in the CSP domain (D). Numerical differences between groups per CSP component are provided.

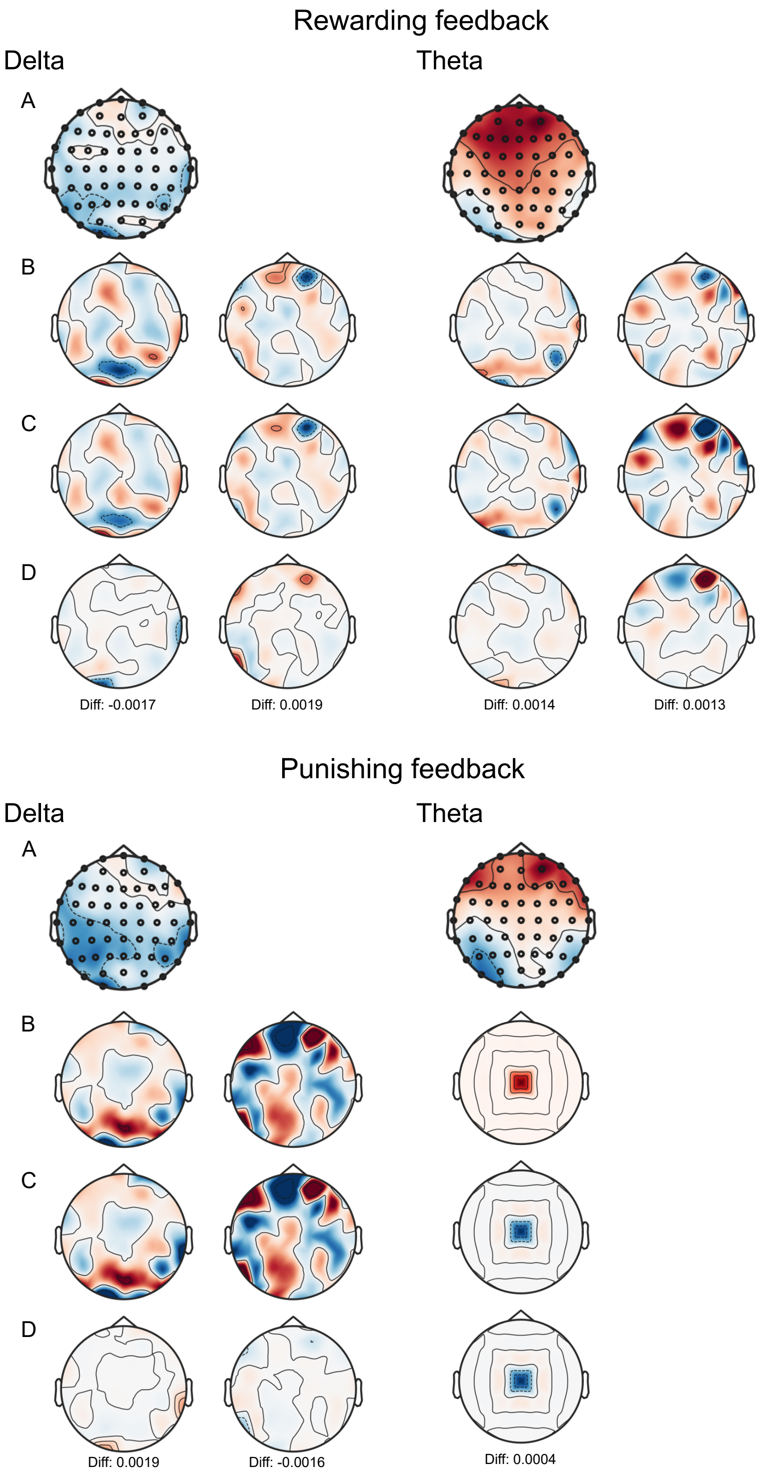

Supplementary Figure S 3. Anxiety vs control group differences. Difference between anxiety and control groups in the frequency domain, before CSP transformation (A). CSP spatial patterns for the anxiety group (B). CSP spatial pattern for the control group (C). Difference between anxiety and control groups in the CSP domain (D). Numerical differences between groups per CSP component are provided.

Distribution of 10x10 CV classification metrics' scores

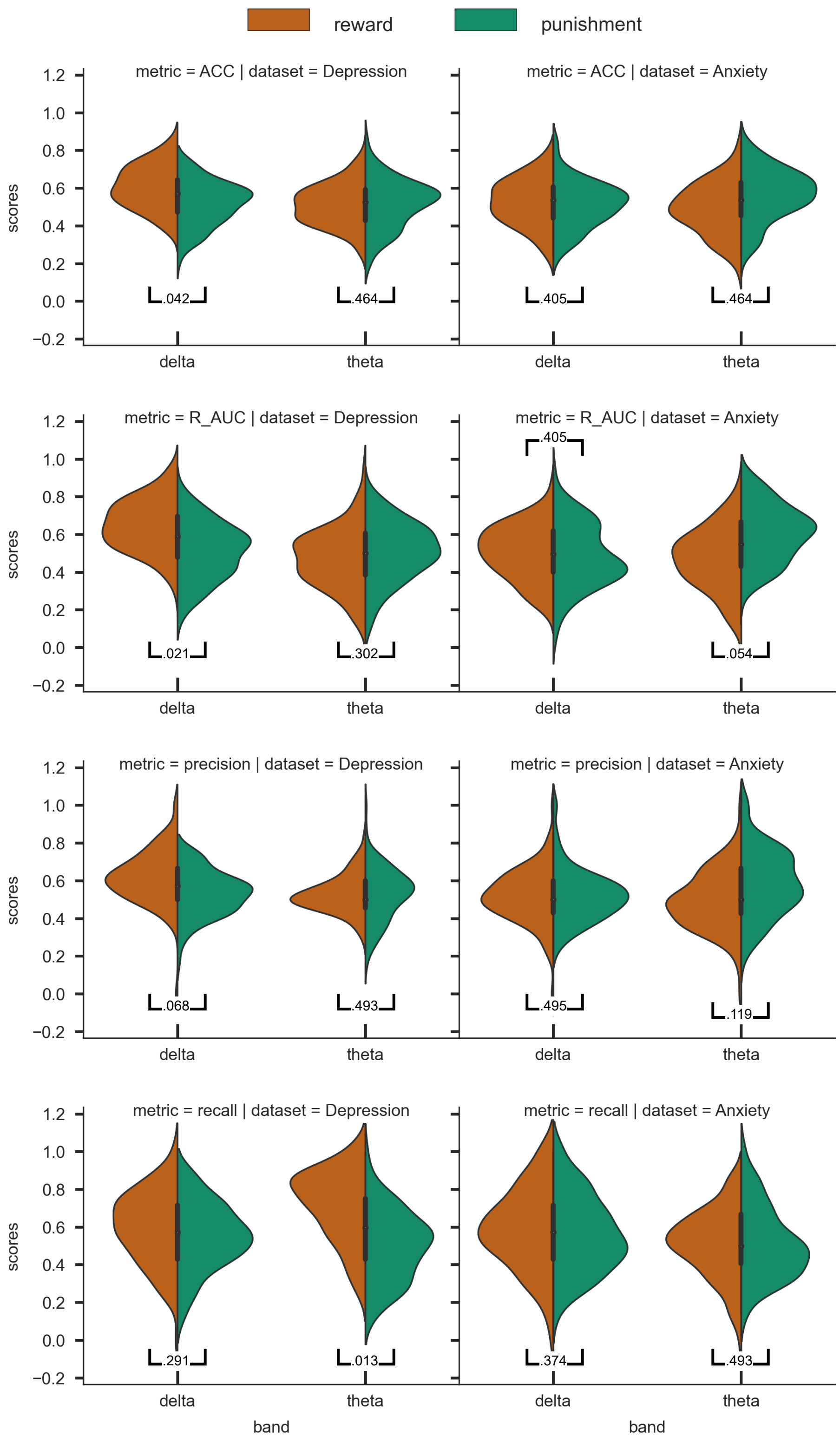

Supplementary Figure S 4. Distribution of 10x10 CV classification metrics' scores for estimated models. Results of significance tests on differences between models are provided.

Exploratory ERP analyses with P3 amplitude control

To investigate the effect of P3 amplitude on the ERP results we conducted an additional exploratory analysis with control for P3 amplitude. The FRN/RewP and P3 amplitudes served as independent variables and group membership was the dependent variable. Similarly to the main ERP analyses performed, SVR served as a classifier. Table 1 shows the results of the exploratory analysis. None of the created models was significant, however, adding P3 information to the model seems to improve the model's performance for depression classification. This is consistent with the main results obtained, indicating that the depression and control groups are well separable based on delta-P3 activity.

Supplementary Table S 1. Detailed results of depression and anxiety models based on RewP and FRN with control on P3 mean amplitude. Findings with *p*-value below 0.05 are shown in bold.

	Depression						Anxiety					
	Train ACC	ACC	*p*-value	R-AUC	Recall	Precision	Train ACC	ACC	*p*-value	R-AUC	Recall	Precision
RewP + P3	.68	.55	.163	.56	.56	.58	.62	.51	.469	.47	.44	.50
FRN + P3	.58	.56	.127	.54	.60	.58	.50	.50	.352	.45	.05	.20

Note. FP = positive/reward feedback; FN = negative/punishment feedback; RewP = reward positivity; FRN = feedback-related negativity; P3 = mean amplitude at FCz in 300 - 400 ms time-window after feedback; ACC = balanced accuracy; R-ACC = ROC AUC. With the exception of Train ACC, all scores reported are the average of 3-fold cross-validation. Train ACC stands for balanced accuracy score estimated on the entire dataset, without cross-validation.
